# Supplementary material for: Personal Exposure to Black Carbon, Particulate Matter and Nitrogen Dioxide in the Paris Region Measured by Portable Sensors Worn by Volunteers
Source: Toxics. 2022 Jan 11;10(1):33. doi: 10.3390/toxics10010033 (PMC8779195; doi:10.3390/toxics10010033)
Supplement: Supplementary file 1 [file toxics-10-00033-s001.zip › toxics-1504463-supplementary.pdf]

toxics

MDPI

Supplementary Materials: Personal Exposure to BC, PM and NO_2 in the Paris Region Measured by Portable Sensors Worn by Volunteers

Baptiste Languille, Valérie Gros, Bonnaire Nicolas, Cécile Honoré, Anne Kaufmann and Karine Zeitouni

S1. IPI Results for BC, NO_2 and PM Sensors.

Table S1.1. IPI results for the AE51 (BC sensors) from the spring assessment campaign. Mean: mean concentration (ng·m^{-3}); Match: match score; RMSE: root mean squared error (ng·m^{-3}); *r*: Pearson correlation coefficient; *t*: Kendall correlation coefficient; *S*: Spearman correlation coefficient; Pres: presence parameter; LFE: low frequencies energy parameter; IPI: integrated performance index.

	#	Mean	Match	RMSE	*r*	*t*	*S*	Pres	LFE	IPI
BC	A1	1001	0.84	334	0.79	0.57	0.74	0.99	0.98	0.80
	A2	976	0.86	302	0.82	0.61	0.79	0.99	0.98	0.82
	A3	1023	0.85	320	0.82	0.62	0.80	0.99	0.99	0.82
	A4	1054	0.83	378	0.80	0.58	0.75	0.95	0.98	0.79
	A5	1035	0.83	397	0.75	0.54	0.72	0.98	0.97	0.77
	A6	1020	0.87	307	0.84	0.65	0.83	0.99	0.99	0.84

Table S1.2. IPI results for the Cairsens (NO_2 sensors). Mean: mean concentration (ppb); Match: match score; RMSE: root mean squared error (ppb); *r*: Pearson correlation coefficient; *t*: Kendall correlation coefficient; *S*: Spearman correlation coefficient; Pres: presence parameter; LFE: low frequencies energy parameter; IPI: integrated performance index.

	#	Mean	Match	RMSE	*r*	*t*	*S*	Pres	LFE	IPI
	C1	49	0.65	19	0.76	0.58	0.75	1.00	0.99	0.76
	C2	39	0.62	13	0.78	0.59	0.77	1.00	0.99	0.77
	C3	44	0.55	16	0.75	0.57	0.75	1.00	0.98	0.75
	C4	48	0.57	17	0.75	0.56	0.73	1.00	1.00	0.75
	C5	37	0.68	14	0.75	0.57	0.75	0.94	0.99	0.76
	C6	44	0.60	15	0.77	0.57	0.75	1.00	0.99	0.76
	C7	45	0.68	14	0.76	0.57	0.74	1.00	1.00	0.78
NO_2	C8	40	0.62	13	0.75	0.56	0.73	1.00	0.99	0.76
	C9	34	0.67	15	0.73	0.55	0.71	1.00	0.99	0.75
	C10	38	0.65	13	0.76	0.57	0.74	1.00	0.99	0.77
	C11	33	0.65	14	0.75	0.57	0.74	1.00	0.99	0.76
	C12	38	0.64	13	0.78	0.59	0.76	1.00	0.99	0.77
	C13	40	0.68	13	0.78	0.59	0.77	1.00	0.99	0.78
	C14	39	0.69	12	0.79	0.60	0.77	1.00	0.99	0.79
	C15	36	0.68	13	0.78	0.59	0.76	1.00	0.99	0.78

Table S1.3. IPI results for the Canarin ($PM_{2.5}$ sensors) from the spring assessment campaign. Mean: mean concentration (μg·m^{-3}); Match: match score; RMSE: root mean squared error (μg·m^{-3}); *r*: Pearson correlation coefficient; *t*: Kendall correlation coefficient; *S*: Spearman correlation coefficient; Pres: presence parameter; LFE: low frequencies energy parameter; IPI: integrated performance index.

	#	Mean	Match	RMSE	*r*	*t*	*S*	Pres	LFE	IPI
$PM_{2.5}$	F1	17	0.23	7	0.88	0.74	0.89	0.79	1.00	0.73
	F2	16	0.29	7	0.84	0.70	0.86	0.80	1.00	0.72
	F3	16	0.38	6	0.86	0.72	0.87	0.81	1.00	0.75
	F4	16	0.27	7	0.87	0.74	0.89	0.80	1.00	0.74
	F5	15	0.26	6	0.76	0.60	0.78	0.81	1.00	0.68
	F6	15	0.70	5	0.84	0.69	0.84	0.62	1.00	0.76
	F7	1	0.16	10	0.00	0.06	0.08	0.72	0.88	–1.10
	F8	16	0.28	6	0.86	0.71	0.87	0.80	1.00	0.73
	F9	15	0.19	6	0.86	0.71	0.86	0.77	1.00	0.72
	F10	14	0.24	5	0.80	0.65	0.81	0.81	1.00	0.70
	F11	14	0.25	5	0.86	0.73	0.88	0.72	1.00	0.73
	F12	14	0.35	5	0.87	0.73	0.87	0.79	1.00	0.75
	F13	15	0.28	5	0.86	0.71	0.86	0.79	1.00	0.73
	F14	16	0.27	8	0.73	0.68	0.84	0.78	1.00	0.69

S2. Sensor Distribution and Association with Volunteer

Table S2.1. Sensor distribution among volunteers during the spring campaign. The sensors are identified by their serial number. * : volunteers that participated to several campaigns. - : sensor used but for which data was corrupted or unusable.

**Volunteer**	**AE51**	**Cairsens**	**Canarin**
AA		-	7
AB		-	15
AC*		-	10
AD*	1241	3975	6
AE		-	16
AF		-	5
AG	1248	-	9
AH	0114	3971	1
AI*		3954	3
AJ		3977	-
AK		3974	14
AL		3978	17
AM	1242	3983	4
AN		-	2
AO	1247	-	-
AP		-	12

Table S2.2. Sensor distribution among volunteers during the autumn campaign. The sensors are identified by their serial number.

Volunteer	AE51	Cairsens	Canarin
AC*		3749	9
AD*	1241	3975	6
AI		3983	5
AQ	1242	3978	7
AR		3755	14
AS	1244	3754	2
AT		3756	13
AU	1247	3750	8
AV	0114	3972	10
AW		3971	4
AX		3970	17
AY		3977	11
AZ	1248	3982	15
BA		3974	16
BB		3979	3

Table S2.3. Sensor distribution among volunteers during the winter campaign. The sensors are identified by their serial number.

Volunteer	AE51	Cairsens	Canarin
BD	0114	3983	5
BE	0114	3983	3
BF	0114	3983	3
BG	0114	3983	3
BH	0114	3983	3
BI	0114	3983	3

S3. Environment Contribution to PE.

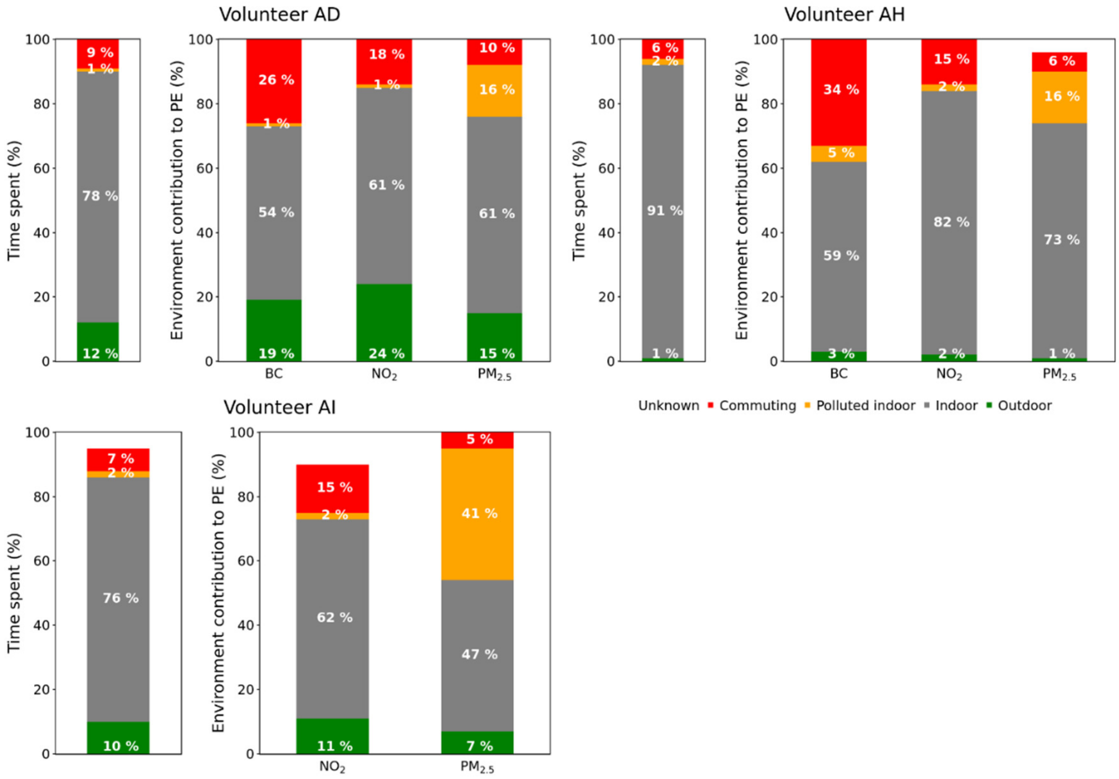

Figure S1. Environment contribution to PE for volunteers AD, AH and AI during the spring campaign.

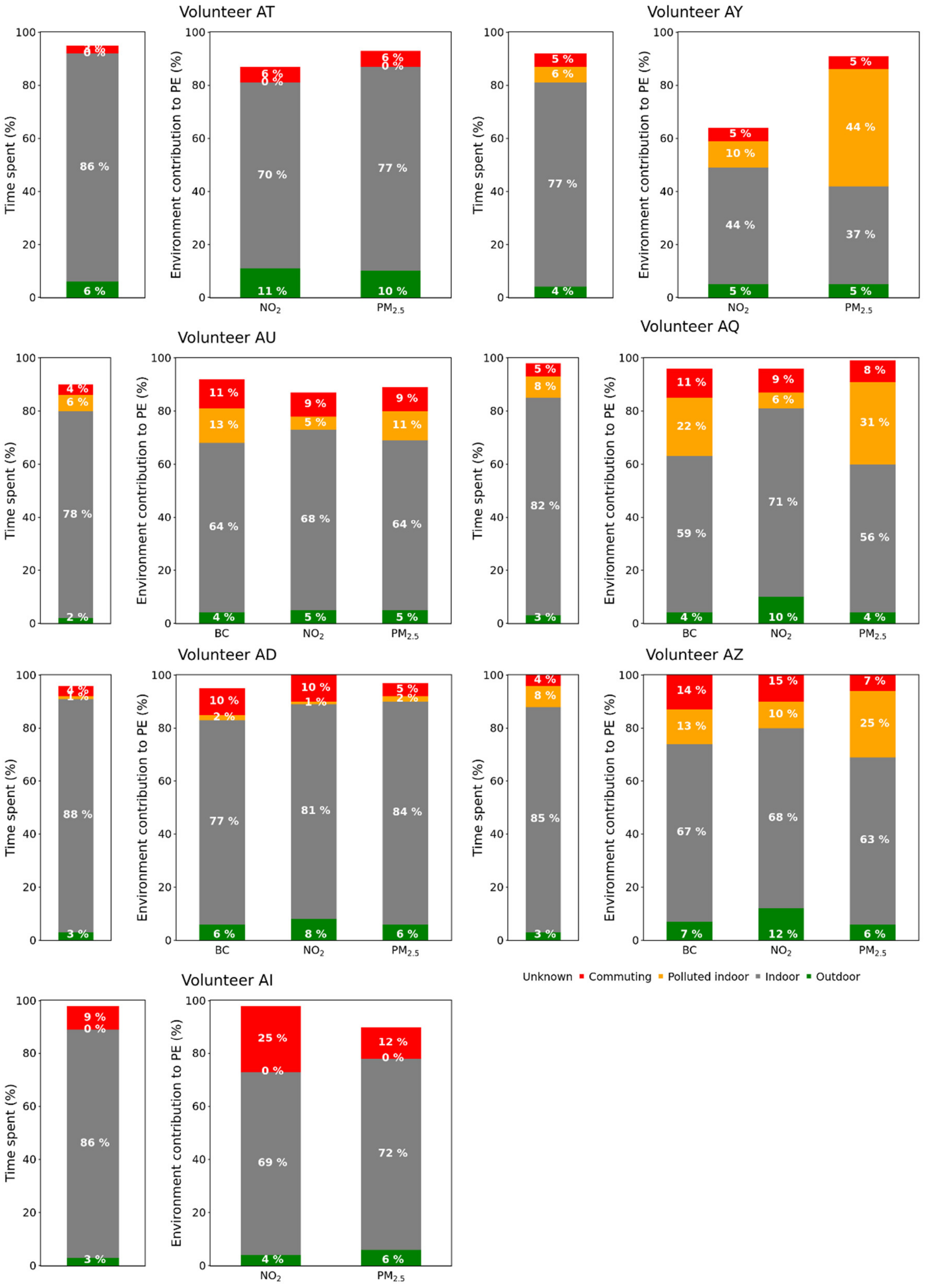

Figure S2. Environment contribution to PE for volunteers AT, AY, AU, AQ, AD, AZ and AI during the autumn campaign.

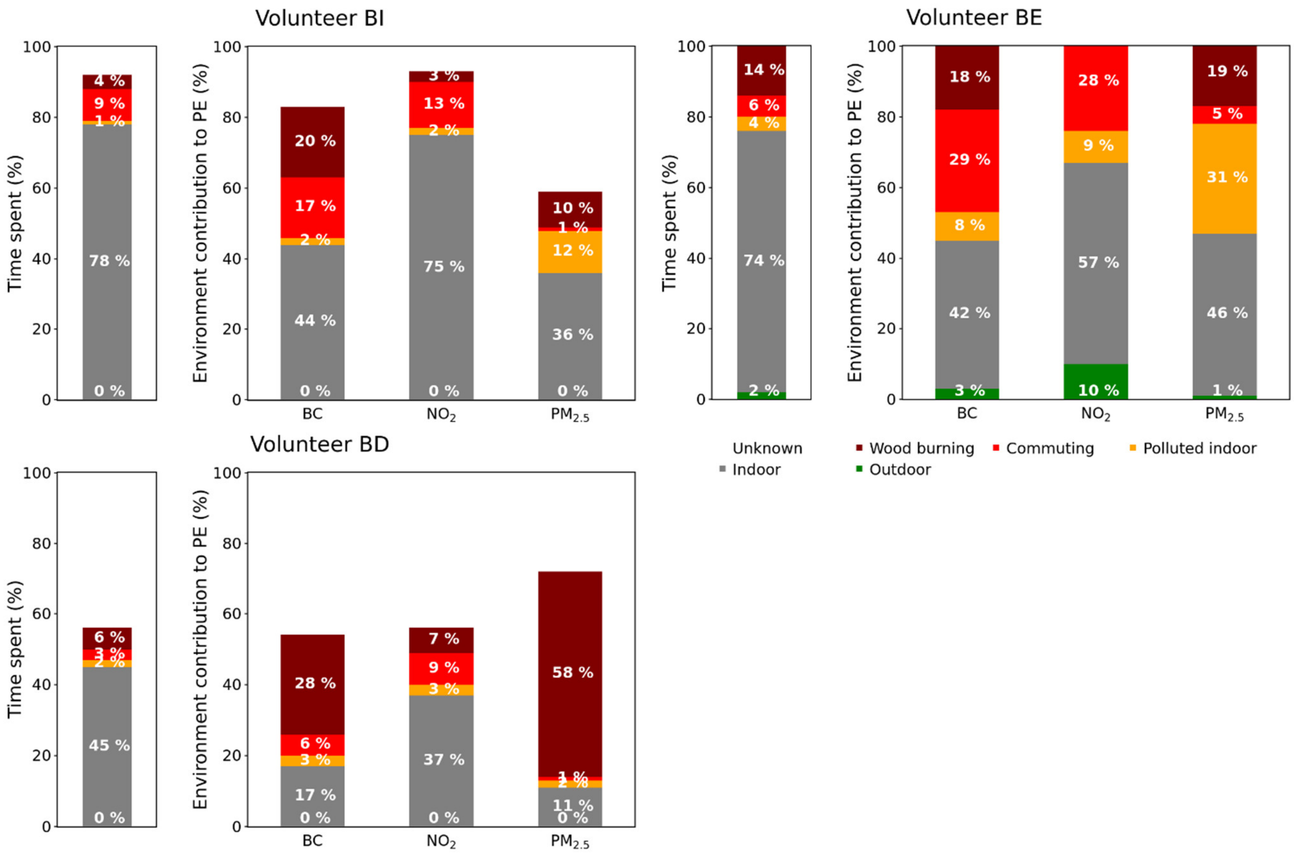

Figure S3. Environment contribution to PE for volunteers BI, BE and BD during the winter campaign.

4. Correlation Matrices and Scatter plots between PE Measured by Sensors and Urban Background Concentrations Monitored by Fixed Stations for Each Campaign for the 1-h Averaged Data Set.

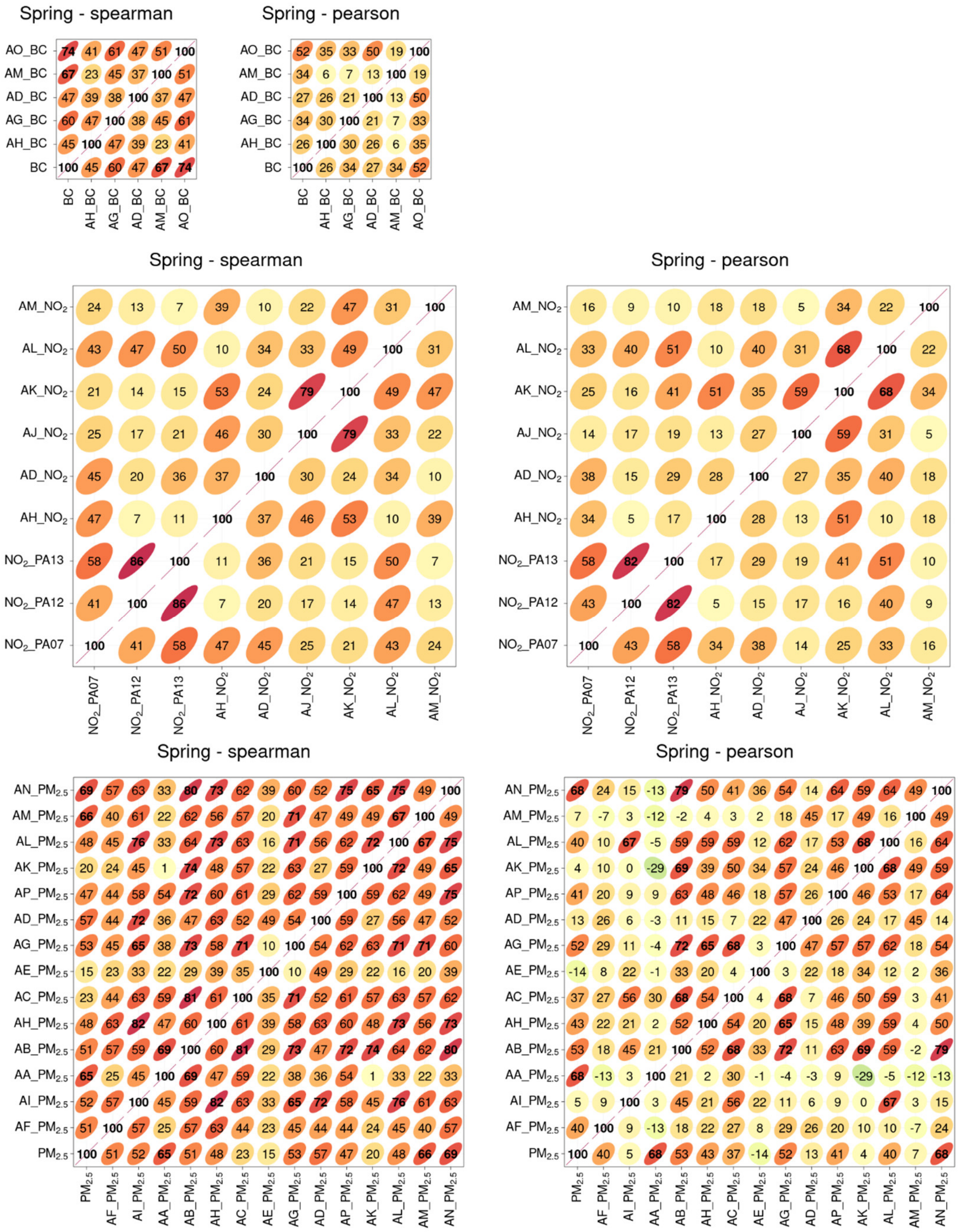

Figure S4. Correlation matrices between PE measured by sensors and urban background concentrations monitored by fixed stations for the spring campaign (1-h averaged data set).

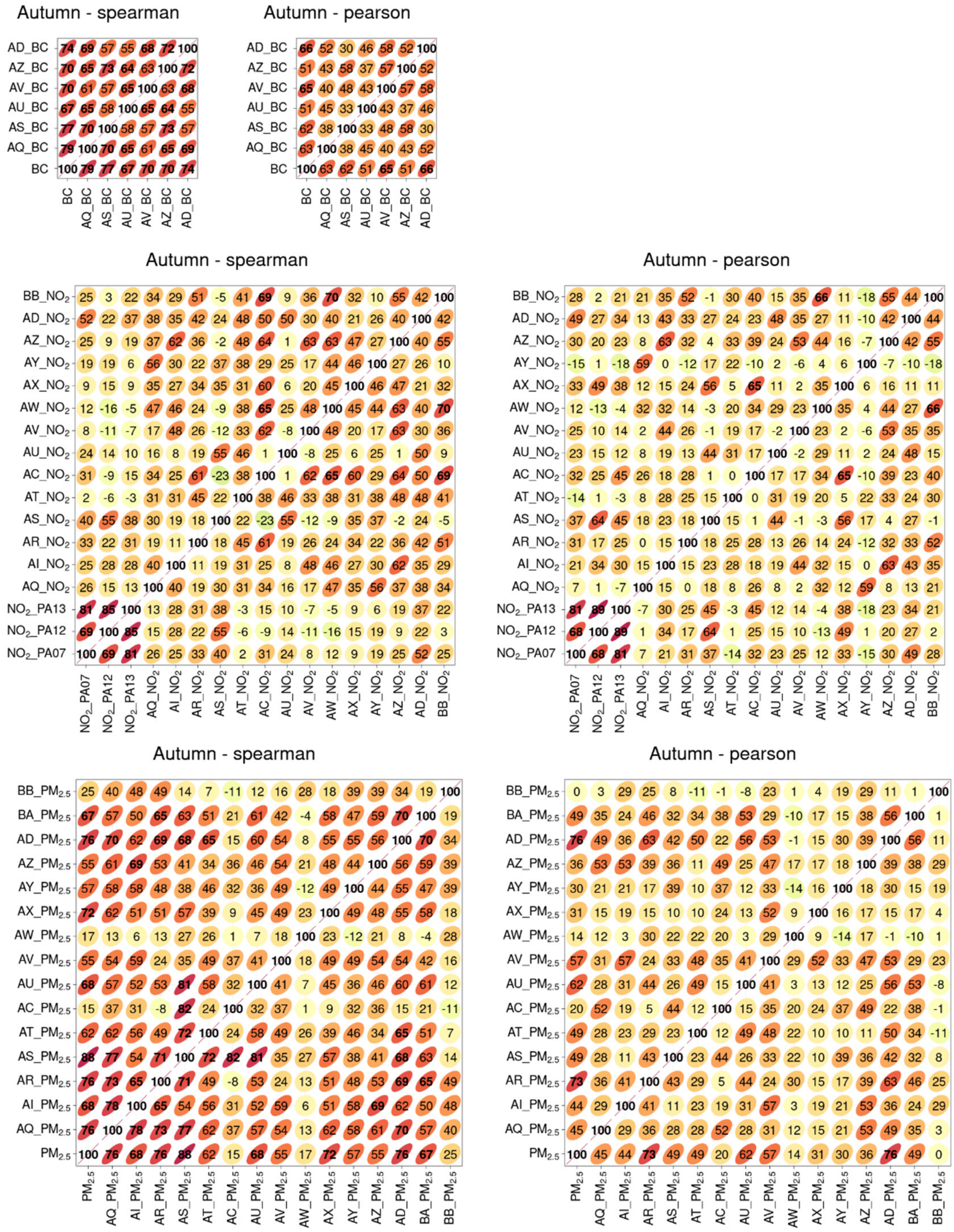

Figure S5. Correlation matrices between PE measured by sensors and urban background concentrations monitored by fixed stations for the autumn campaign (1-h averaged data set).

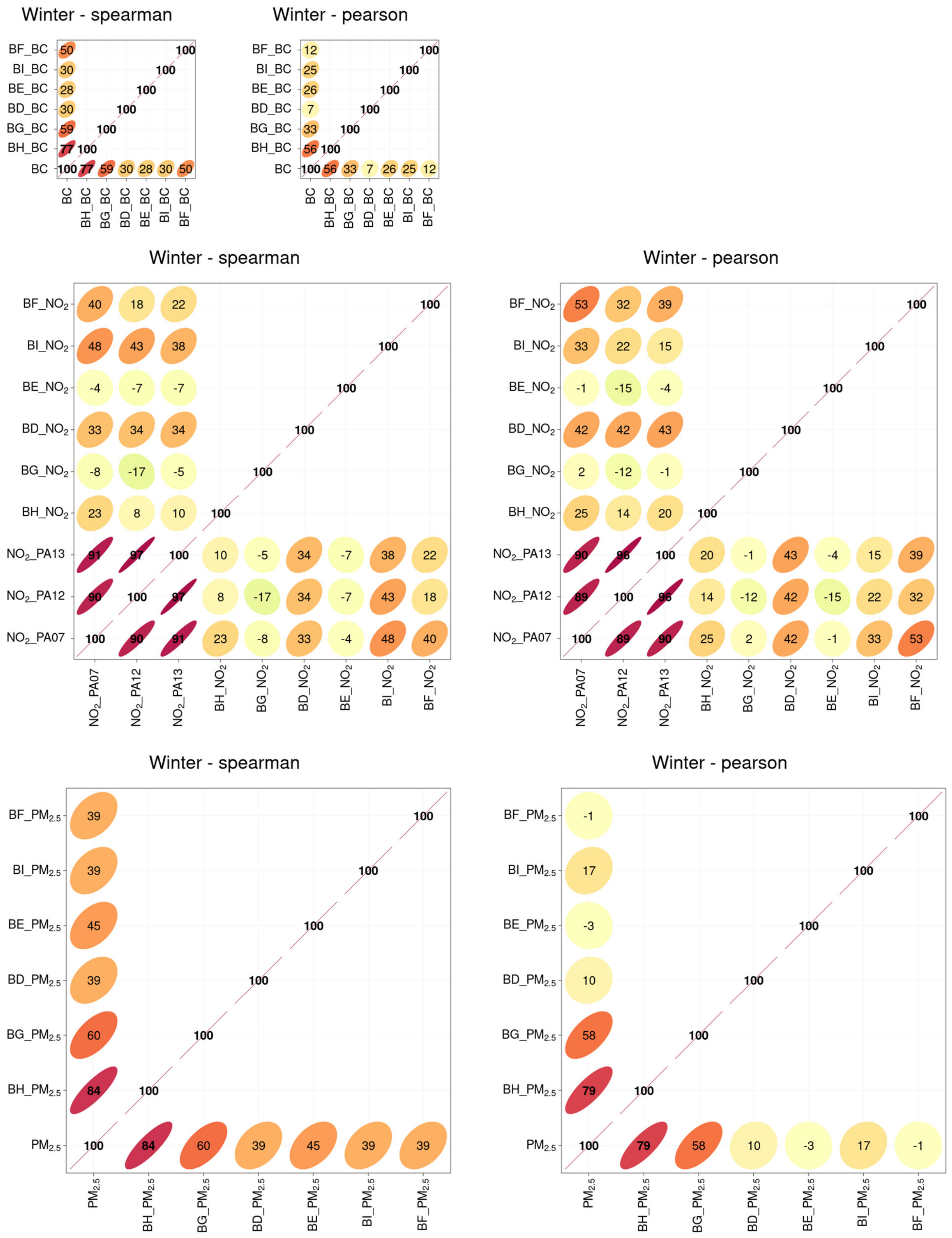

Figure S6. Correlation matrices between PE measured by sensors and urban background concentrations monitored by fixed stations for the winter campaign (1-h averaged data set).
